# The effectiveness of an on-line training program for improving knowledge of fire prevention and evacuation of healthcare workers: A randomized controlled trial

**DOI:** 10.1371/journal.pone.0199747

**Published:** 2018-07-05

**Authors:** Paul H. Lee, Baoguo Fu, Wangting Cai, Jingya Chen, Zhenfei Yuan, Lifen Zhang, Xiuhong Ying

**Affiliations:** 1 School of Nursing, Hong Kong Polytechnic University, Kowloon, Hong Kong; 2 Department of Disaster Nursing, Sichuan University—The Hong Kong Polytechnic University Institute for Disaster Management and Reconstruction, Chengdu, China; 3 The Second Clinical Medical College, Yangtze University, Jingzhou, China; University of Newcastle, AUSTRALIA

## Abstract

**Background:**

Hospitals are vulnerable to fires and the evacuation process is challenging. However, face-to-face fire prevention and evacuation training may take healthcare workers’ time away from patient care; therefore, effective on-line training may be warranted. We carried out and examined the effectiveness of an on-line education and training of fire prevention and evacuation training for healthcare workers in China by a randomized controlled trial using convenience sampling from five public hospitals in China.

**Methods:**

A total of 128 participants were recruited between December 2014 and March 2015. The authors built a webpage that included the informed consent statement, pre-test questionnaire, video training, and post-test questionnaire. After completing the pre-test questionnaire, participants were randomly assigned to watch the intervention video (basic response to a hospital fire) or the control video (introduction to volcanic disasters). A 45-item questionnaire on knowledge of fire prevention and evacuation was administered before and after the video watching. This questionnaire were further divided into two subscales (25-item generic knowledge of fire response and 20-item hospital-specific knowledge of fire prevention and evacuation). One point was awarded for each correct answer.

**Results:**

Half of the participants (n = 64, 50%) were randomized into the intervention group and the remaining 64 (50%) were randomized into the control group. For generic knowledge of fire prevention and evacuation, those in the intervention group improved significantly (from 16.16 to 20.44, *P* < 0.001) while the scores of those in the control group decreased significantly (from 15.27 to 13.70, *P* = 0.03). For hospital-specific knowledge of fire prevention and evacuation, those in the intervention group (from 10.75 to 11.33, *P* = 0.15) and the control group (from 10.38 to 10.16, *P* = 0.54) had insignificant change. For total score, those in the intervention group improved significantly (from 26.91 to 31.77, *P* < 0.001) while those in the control group decreased insignificantly (from 25.64 to 23.86, *P* = 0.07). After the intervention, the difference between the scores of the intervention group and the control group on all three knowledge areas of fire prevention and evacuation (generic, hospital-specific, and total) were significant (all *P*s < 0.05).

**Conclusions:**

An on-line fire training program delivered via educational video can effectively improve healthcare workers’ knowledge of fire prevention and evacuation.

**Trial registration:**

Clinicaltrials.gov NCT02438150

## Introduction

Fire is defined as uncontrolled burning in time and space releasing heat that may result in disaster [1]; if the fire is not immediately handled properly, the consequences will be very serious. Flammable, explosive, and other combustible materials will cause the fire to grow, personnel and material will be damaged, and we can imagine that tall buildings may fall. One of the building types vulnerable to a fire disaster is a hospital. Hospitals are more vulnerable to fire than many buildings as they usually store more flammable materials than other buildings. For example, there are many medical gas cylinders stored in a hospital, and ignition of these cylinders will cause fire [2]. In addition to the aforementioned risks, there are many critically ill patients in hospitals who are difficult to transport, so the evacuation procedure is challenging and the consequences are even more serious when a fire occurs [3]. Even when the fire is not serious and evacuation is not required, there exist other environmental emergencies, such as power failure, that need immediate response by healthcare workers [4]. Reports of domestic and international hospital fires in recent years [5] have brought this issue to general attention. The National Health and Family Planning Commission of China requires all medical and health institutions to pay extra attention to personnel evacuation and transfer during the emergency handling of an accident, as they consider emergency handling to be a key element to avoid and reduce casualties to the greatest extent [6]. In view of these national requirements and the seriousness of the consequences of a hospital fire, many hospitals are regularly carrying out fire-related training, but the training effectiveness and method are also worth examining.

Training for fire prevention and handling has frequently been reported in the literature, but most of this training was developed for firefighters and not for healthcare workers [7, 8]. Owing to the busy schedule of healthcare workers, fire drills in hospitals often struggle to achieve a high attendance rate, and most of this training focuses on case studies, face-to-face lectures, examinations, and developing plans [9]. In addition, most hospitals did not offer regular fire drills [10], which may be preferred by health workers as they could focus on patient care rather than the fire drill. There are many existing online fire training materials available (a search in Google yielded more than 700,000 results), but their reliability is questionable, and most physicians prefer trustworthy on-line resources over general search engines [11]. This result supported the need to develop reliable online fire training materials. On-line training, defined as “the study and ethical practice of facilitating learning and improving performance by creating, using, and managing appropriate technological processes and resources” [12], provides healthcare workers with a flexible, convenient, effective training method to master knowledge of fire prevention, handling, self-rescue, and appropriate patient evacuation methods. Online training has the advantages of being convenient, flexible, affordable, and efficient [13]. At present, hospital management and first-line healthcare workers are busy dealing with day-to-day responsibilities, and organizing fire drills and training is often subject to the constraints of time and place. Thus, on-line training is the perfect solution as it can be conducted at any time and any place [14]. With the development of information technology, utilizing computers for training is becoming increasingly popular, and online training for fire response and related knowledge has promising benefits. On-line training programs and courses for healthcare workers are becoming increasingly popular worldwide [15]. On-line training in other fields has been shown to be effective [14, 16], but online fire training for healthcare workers is sparse. Therefore, we carried out and examined the effectiveness of an on-line knowledge of fire prevention and evacuation training for healthcare workers in China.

## Methods

### Participants

Participants were recruited from five public hospitals in Chengdu (Sichuan Provincial People’s Hospital and West China Hospital of Sichuan University), Kunming (Second Affiliated Hospital of Kunming Medical University), Jingzhou (Jingzhou Central Hospital), and Fuzhou (First Affiliated Hospital of Fujian Medical University) in China. All five hospitals were under the same classification (3A: provincial or national level hospitals with >500 beds) and did not provide regular fire training to healthcare workers. Participants were recruited between December 2014 and March 2015 by convenience sampling by the authors, and those who agreed to participate were e-mailed with a username and password to the webpage for video viewing, together with the information of the study. Only those who had more than one year of experience working in their current position were eligible. An email reminder was provided if the participants had not logged into the webpage in 2 weeks.

### Ethical consideration

Informed consent was given through the webpage, and this study was approved by the Human Subjects Ethics Sub-committee of the Hong Kong Polytechnic University (HSEARS20141020002). The protocol and CONSORT 2010 checklist can be found in the supplementary materials S1 and S2 Files.

### Intervention and control

**Fig 1:**
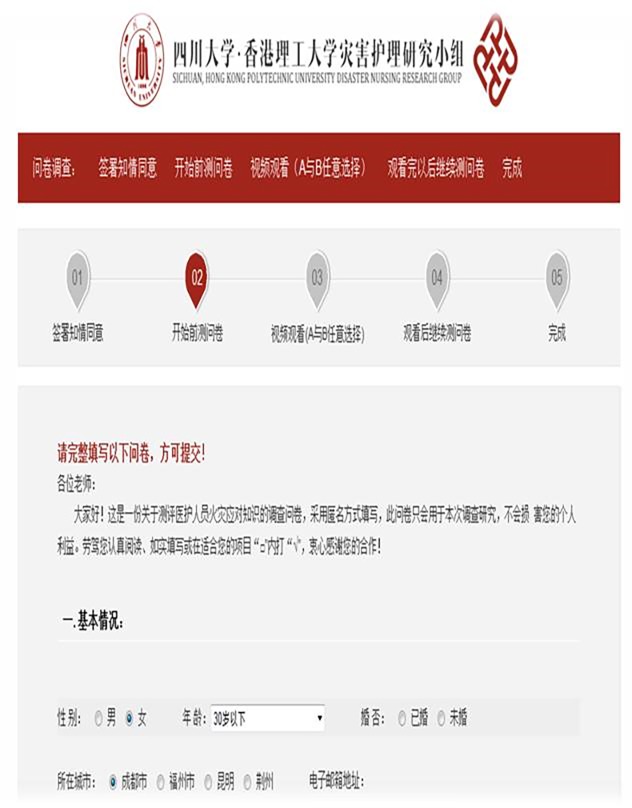
Index page of the intervention webpage.

The authors built a webpage (www.puidmr.com) that includes the informed consent agreement, pre-test questionnaire, video training, and post-test questionnaire. Fig 1 shows a screenshot of the index page, and S1 Fig shows a screenshot of the informed consent and first page of the questionnaire. Participants had to respond to all questions before they could proceed to the next step. After completing the pre-test questionnaire, participants were randomly assigned by the webpage (using the Math.random() function) with a 1:1 ratio to watch one of the two videos. Those in the intervention group watched a 14-minute video showing the basic response to a hospital fire and patient evacuation methods from the National Fire Protection Association (permission obtained) with Chinese subtitles written by the authors. Those in the control group watched a 6-minute video introducing volcanic disasters. We chose a shorter video for the control group, as we believed that the compliance among the control group would be reduced if the video were long. After completing the post-test questionnaire, those in the control group were allowed to watch the intervention video on the webpage. To explore the possible bugs in the webpage, a pilot trial on 20 subjects was conducted before the start of the intervention. The content remained unchanged during the intervention period.

### Data collection

The questionnaire was used to collect data on demographic characteristics and socio-economic status, and it also consisted of 45 items on knowledge of fire prevention and evacuation and was administered before and after the video watching. The questionnaire was administered in Chinese and the English translation can be found in S3 File. The items on knowledge of fire prevention and evacuation can be further divided into two subscales; the first subscale consists of 25 items on generic knowledge of fire response, including prevention and disposal (for example, “When a fire occurs, you should take the nearest elevator to evacuate.”), and the second subscale consists of 20 items on hospital-specific knowledge related to responding to a hospital fire and selecting equipment under a fire emergency in a hospital (for example, “bed-bound patients should be evacuated by bed linen.”). One point was awarded for each correct answer and no point was awarded for each wrong answer.

### Sample size estimation

Assuming that the Cohen’s *d* effect size of the intervention is 0.5 (similar to other online trainings [14]), 64 participants per group were adequate could achieve a power of 0.8 for 5% level of significance.

### Statistical analysis

The data analyst was blinded to the group assignment. Descriptive statistics (mean, SD, frequency, and percentage) were used to describe the study participants. The associations between knowledge of fire prevention and evacuation scores and baseline descriptive statistics were evaluated using ANOVA, and eta-squared was used to quantify their effect sizes. An eta-squared of >0.02 was regarded as at least a small effect size [17]. The within group and between group knowledge of fire prevention and evacuation scores were compared using paired-sample *t* tests and independent-sample *t* tests respectively. Linear regressions were used to evaluate the effectiveness of the intervention, treating the post-test fire knowledge scores as dependent variables and group allocation and pre-test knowledge of fire prevention and evacuation scores as independent variables. For the adjusted analysis, variables with an eta-squared of >0.02 with knowledge of fire prevention and evacuation scores were included as covariates [18]. The analyses were conducted using SPSS 22.0.

## Results

### Participants

**Fig 2:**
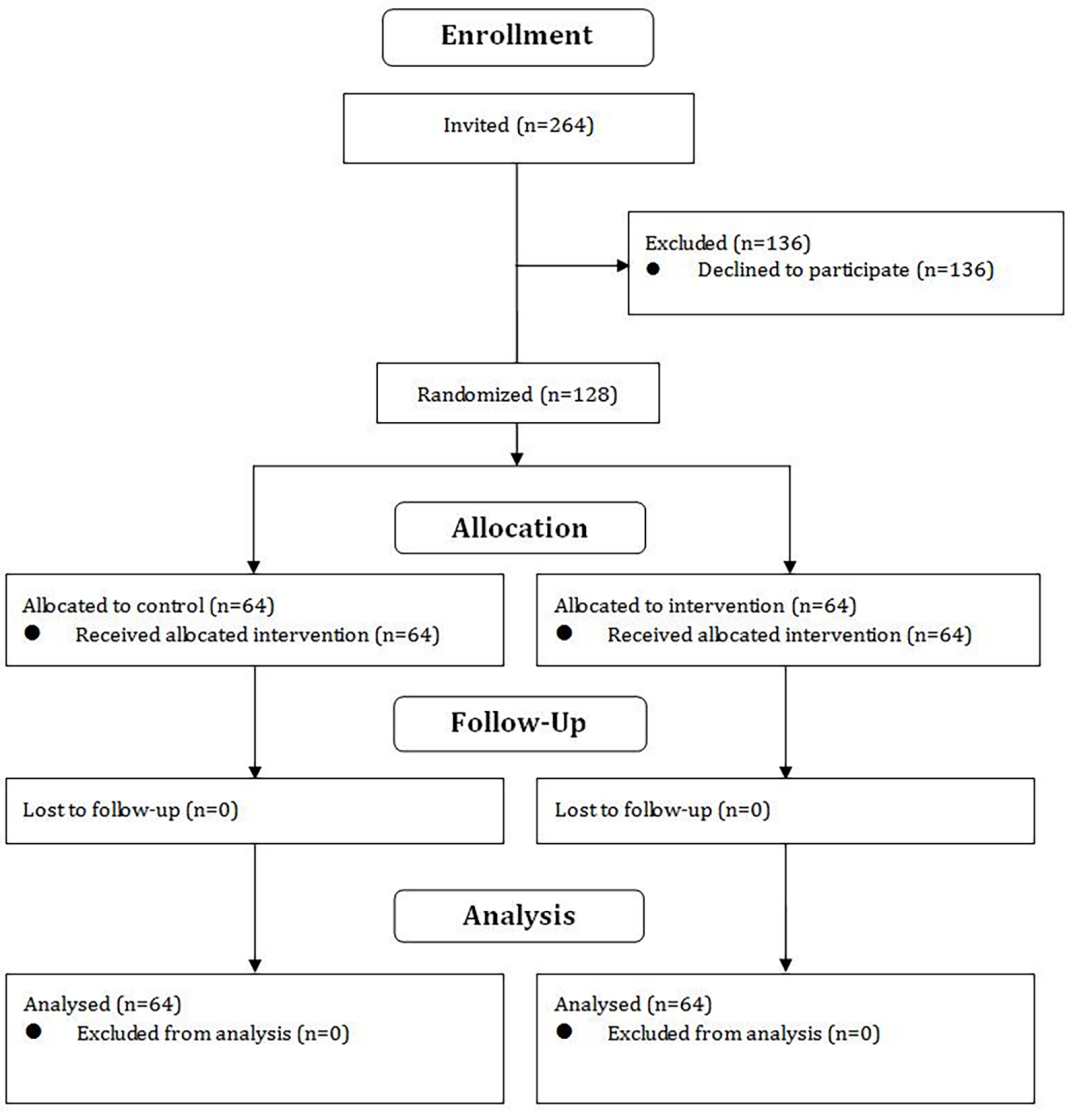
CONSORT diagram.

A total of 264 healthcare workers were invited and 128 (48.5%) agreed to participate (Fig 2). Half of them (n = 64, 50%) were randomized into the intervention group, and the remaining 64 (50%) were randomized into the control group. All of them completed the pre-test and pro-test questionnaire.

### Descriptive statistics

Table 1 shows the descriptive statistics of the participants. A majority of them were female (82.8%) and aged 29 years or younger (61.7%). Around one-third participated in the fire prevention training and fire evacuation training (32.0% and 38.3%, respectively) organized by their hospital. Surprisingly, less than one-fourth (24.2%) of the participants perceived their knowledge of fire prevention and evacuation as very good or good. Note that nearly all (84.4%) prefer live demonstration over online training.

**Table 1:** Descriptive statistics of the study participants (n = 128).

	Intervention group	Control group	*P*-value
	n = 64	n = 64	
**Sex**			.26
Male	12 (19%)	7 (11%)	
Female	52 (81.%)	54 (89%)	
**Age**			.39
29 or younger	36 (56%)	43 (67%)	
30–39	23 (36%)	16 (25%)	
40–49	4 (6%)	5 (8%)	
50 or above	1 (2%)	0 (0%)	
**Marital status**			.85
Married	37 (64%)	36 (62%)	
Others	21 (36%)	22 (38%)	
**City**			.001
Chengdu	24 (38%)	26 (41%)	
Kunming	27 (42%)	10 (16%)	
Jingzhou	13 (20%)	21 (33%)	
Fuzhou	0 (0.0%)	6 (9%)	
Missing	0 (0.0%)	1 (2%)	
**Working experience**			.07
Less than 3 years	15 (23%)	25 (39%)	
3–5 years	10 (16%)	10 (16%)	
6–9 years	22 (34%)	10 (16%)	
10 years or more	17 (27%)	19 (30%)	
**Job**			.43
Nurse	53 (83%)	58 (91%)	
Doctor	7 (11%)	4 (6%)	
Therapist	4 (6%)	2 (3%)	
**Monthly salary in CNY (¥)**			.21
1–3000	17 (26%)	11 (17%)	
3001–5000	29 (45%)	27 (42%)	
5001–8000	14 (22%)	22 (34%)	
8001 or above	2 (3%)	4 (6%)	
Missing	2 (3%)	0 (0.0%)	
**Religion**			.73
Yes	5 (8%)	4 (6%)	
No	59 (92%)	60 (94%)	
**Education**			.37
Secondary	0 (0.0%)	1 (2%)	
Tertiary non-degree	11 (17%)	14 (22%)	
Bachelor degree	44 (69%)	44 (69%)	
Master	9 (14%)	4 (6%)	
Doctorate	0 (0.0%)	1 (2%)	
**Participated in fire prevention training organized by hospital**			1
Yes	20 (32%)	20 (32%)	
No	43 (68%)	43 (68%)	
**Participated in fire evacuation training organized by hospital**			.86
Yes	25 (39%)	24 (38%)	
No	39 (61%)	40 (63%)	
**Perceived knowledge of fire prevention and evacuation**			.76
Very good	1 (2%)	2 (3%)	
Good	16 (25%)	12 (19%)	
Natural	32 (50%)	36 (56%)	
Poor	15 (23%)	14 (22%)	
**Preferred mode of training**			.34
Face-to-face	0 (0%)	2 (3%)	
Online	7 (11%)	9 (14%)	
Live demonstration	56 (88%)	52 (81%)	
Manual-reading	1 (2%)	0 (0%)	
Missing	0 (0%)	1 (2%)	
**Fire prevention protocol exists in hospital**			.86
Yes	31 (48%)	30 (47%)	
No	33 (52%)	34 (53%)	

### Associations between baseline characteristics and knowledge of fire prevention and evacuation

Table 2 shows the associations between baseline characteristics and the knowledge of fire prevention and evacuation scores of the participants. The mean scores for the generic knowledge of fire prevention and evacuation, hospital-specific knowledge of fire prevention and evacuation, and the total scores were 15.71 (4.00), 10.56 (2.17), and 26.27 (5.25), respectively. This finding shows that the participants on average had better generic knowledge of fire prevention and evacuation than hospital-specific knowledge of fire prevention and evacuation. Those who were aged 30–39 years, had a higher education level, participated in fire evacuation training, had higher perceived knowledge of fire prevention and evacuation, preferred live demonstration training, and worked in hospitals with a fire prevention protocol had higher levels of knowledge of fire prevention and evacuation.

**Table 2:** Association between baseline characteristics and knowledge of fire prevention and evacuation of the study participants (n = 128).

	Generic knowledge of fire prevention and evacuation (0–25)	Hospital-specific knowledge of fire prevention and evacuation (0–20)	Total (0–45)
	Mean (SD)	Mean (SD)	Mean (SD)
**Sex**			
Male	17.11 (2.47)	10.53 (1.95)	27.63 (3.68)
Female	15.52 (4.16)	10.59 (2.24)	26.11 (5.45)
*P*-value	.11	.90	.25
Eta squared	0.02	0.0001	0.01
**Age**			
29 or younger	14.76 (4.29)	10.41 (2.13)	25.16 (5.56)
30–39	17.90 (2.35)	10.85 (2.29)	28.74 (3.70)
40–49	14.78 (3.89)	10.78 (2.22)	25.56 (5.29)
50 or above	14 (NA)	10 (NA)	24 (NA)
*P*-value	.001	.75	.005
Eta squared	0.13	0.01	0.10
**Marital status**			
Married	16.16 (3.83)	10.82 (2.19)	26.99 (4.87)
Others	14.67 (4.13)	10.30 (2.13)	24.98 (5.63)
*P*-value	.052	.045	.22
Eta squared	0.03	0.01	0.03
**City**			
Chengdu	15.86 (5.07)	10.66 (2.17)	26.52 (6.39)
Kunming	15.76 (3.00)	10.54 (2.10)	26.30 (3.83)
Jingzhou	15.24 (3.49)	9.97 (2.02)	25.21 (4.78)
Fuzhou	17.17 (2.32)	13.33 (1.63)	30.50 (3.62)
*P*-value	.73	.005	.15
Eta squared	0.01	0.10	0.04
**Working experience**			
Less than 3 years	16.03 (4.04)	10.60 (1.96)	26.63 (5.18)
3–5 years	13.40 (4.74)	10.60 (2.46)	24.00 (6.42)
6–9 years	15.75 (3.88)	10.22 (2.32)	25.97 (5.17)
10 years or more	16.61 (3.25)	10.81 (2.14)	27.42 (4.43)
*P*-value	.03	.74	.12
Eta squared	0.07	0.01	0.05
**Job**			
Nurse	15.68 (4.11)	10.61 (2.24)	26.29 (5.42)
Doctor	16.82 (3.03)	10.00 (1.55)	26.82 (4.05)
Therapist	14.33 (3.27)	10.67 (1.86)	25.00 (4.34)
*P*-value	.46	.67	.79
Eta squared	0.01	0.01	0.004
**Monthly salary in CNY (¥)**			
1–3000	16.14 (3.75)	11.00 (2.02)	27.14 (4.92)
3001–5000	14.89 (4.38)	10.48 (2.28)	25.38 (5.73)
5001–8000	16.17 (3.48)	10.31 (2.08)	26.47 (4.59)
8001 or above	17.83 (4.17)	11.67 (2.07)	29.50 (5.92)
*P*-value	.24	.19	.32
Eta squared	0.04	0.05	0.04
**Religion**			
Yes	15.22 (3.96)	10.89 (2.52)	26.11 (5.42)
No	15.75 (4.02)	10.54 (2.15)	26.29 (5.26)
*P*-value	.71	.64	.92
Eta squared	0.001	0.002	<0.001
**Education**			
Secondary	17 (NA)	13 (NA)	30 (NA)
Tertiary non-degree	13.32 (5.26)	10.52 (2.50)	23.84 (6.59)
Bachelor degree	15.93 (3.52)	10.59 (2.17)	26.52 (4.90)
Master	18.38 (1.76)	10.23 (1.59)	28.62 (3.01)
Doctorate	20 (NA)	11 (NA)	31 (NA)
*P*-value	.002	.81	.048
Eta squared	0.13	0.01	0.07
**Participated in fire prevention training organized by hospital**			
Yes	16.35 (3.47)	10.50 (1.93)	26.85 (4.57)
No	15.34 (4.22)	10.58 (2.30)	25.92 (5.57)
*P*-value	.19	.85	.36
Eta squared	0.01	<0.001	0.01
**Participated in fire evacuation training organized by hospital**			
Yes	17.65 (2.88)	11.24 (2.03)	28.90 (3.98)
No	14.51 (4.14)	10.14 (2.16)	24.65 (5.31)
*P*-value	<0.001	.005	<0.001
Eta squared	0.15	0.06	0.16
**Perceived knowledge of fire prevention and evacuation**			
Very good	19.00 (4.00)	12.33 (0.58)	31.33 (4.04)
Good	17.71 (2.87)	11.21 (2.51)	28.93 (4.05)
Natural	15.26 (4.54)	10.25 (2.17)	25.51 (5.91)
Poor	14.48 (2.65)	10.48 (1.70)	24.97 (3.42)
*P*-value	.005	.11	.003
Eta squared	0.10	0.05	0.10
**Preferred mode of training**			
Face-to-face	13.00 (0.00)	11.50 (0.71)	24.50 (0.71)
Online	10.88 (4.90)	9.69 (2.39)	20.56 (6.21)
Live demonstration	16.44 (3.37)	10.68 (2.15)	27.12 (4.65)
Manual-reading	19 (NA)	10 (NA)	29 (NA)
*P*-value	<0.001	.35	<0.001
Eta squared	0.23	0.03	0.18
**Fire prevention protocol exists in hospital**			
Yes	16.77 (3.67)	10.87 (1.90)	27.64 (4.60)
No	14.75 (4.08)	10.28 (2.37)	25.03 (5.52)
*P*-value	.004	.13	.005
Eta squared	0.06	0.02	0.06

### Knowledge of fire prevention and evacuation before and after the intervention

Table 3 shows the knowledge of fire prevention and evacuation scores of the participants before and after the intervention. For generic knowledge of fire prevention and evacuation, those in the intervention group improved significantly while those in the control group decreased significantly. For hospital-specific knowledge of fire prevention and evacuation, those in the intervention group and the control group had insignificant change. For total score, those in the intervention group improved significantly while those in the control group decreased insignificantly. After the intervention, the differences in all three aspects of knowledge of fire prevention and evacuation scores between the intervention group and control group were significant.

**Table 3:** Knowledge of fire prevention and evacuation of the study participants before and after the intervention (n = 128).

	Intervention group	Control group	*P*-value
	n = 64	n = 64	
**Generic knowledge of fire prevention and evacuation (0–25)**			
Pre-test	16.16 (3.87)	15.27 (4.11)	.21
Post-test	20.44 (2.57)	13.70 (4.90)	<0.001
*P*-value	<0.001	.03	
**Hospital-specific knowledge of fire prevention and evacuation (0–20)**			
Pre-test	10.75 (2.02)	10.38 (2.31)	.33
Post-test	11.33 (1.92)	10.16 (2.32)	.002
*P*-value	.15	.54	
**Total (0–45)**			
Pre-test	26.90 (4.81)	25.64 (5.62)	.17
Post-test	31.77 (3.12)	23.86 (6.52)	<0.001
*P*-value	<0.001	.07	

### Effectiveness of the intervention on knowledge of fire prevention and evacuation

Table 4 shows the results regressing post-test knowledge of fire prevention and evacuation scores with baseline characteristics. The crude and adjusted effects of the intervention were very similar, and results showed that the intervention improved the generic knowledge of fire prevention and evacuation, hospital-specific knowledge of fire prevention and evacuation, and total score by 6.41 (95% CI 4.94, 7.88) points, 0.99 (95% CI 0.22, 1.75) points, and 6.74 (95% CI 4.78, 8.69) points, respectively. These scores correspond to Cohen’s *d* effect sizes of 1.60, 0.46, and 1.28, respectively.

**Table 4:** Crude ^a^ and adjusted ^b^ effects of the intervention (n = 128).

	Difference between intervention and control groups (95% CI)	*P*-value
**Generic knowledge of fire prevention and evacuation (0–25)**		
Crude	6.62 (5.28, 7.96)	<0.001
Adjusted	6.41 (4.94, 7.88)	<0.001
**Hospital-specific knowledge of fire prevention and evacuation (0–20)**		
Crude	1.16 (0.43, 1.89)	.002
Adjusted	0.99 (0.22, 1.75)	.012
**Total (0–45)**		
Crude	7.76 (6.00, 9.51)	<0.001
Adjusted	6.74 (4.78, 8.69)	<0.001

^a^ adjusted for baseline knowledge of fire prevention and evacuation score only.

^b^ adjusted for baseline knowledge of fire prevention and evacuation score and characteristics associated with post-test outcome with eta squared effect size of >0.02.

## Discussion

Our results showed that on-line training is effective in improving healthcare workers’ knowledge of fire prevention and evacuation. While its effect on improving generic knowledge of fire prevention and evacuation was large (Cohen’s *d* = 1.60), its effect on hospital-specific knowledge of fire prevention and evacuation was small-to-medium (*d* = 0.46). Further on-line fire prevention and evacuation training should focus on improving hospital-specific knowledge of fire prevention and evacuation of the participants.

As healthcare workers face various types of hospital disasters, their training should be efficient and short so as not to occupy their valuable working time [19–24]. Furthermore, hospitals should provide training on fire safety through education, examination, and the development of a sense of awareness of fire safety and rescue strategies among healthcare workers [5, 9, 25–30]. Our results supported the use of on-line fire training for healthcare workers. We have shown the effectiveness of video training, and the effectiveness of other formats of on-line fire safety training, such as serious games [31] and augmented reality [32], should also be explored.

On-line fire prevention and evacuation training is most suitable for hospitals that lack regular fire drills and other fire safety training. The use of on-line fire training is supported by the results of our study. On-line fire safety training can also be used as a complement for healthcare workers who are unable to attend fire drills.

On-line training is most suitable for teaching important skills that are not covered in the medical curriculum [33]. The development of further on-line teaching materials with emerging knowledge essential for healthcare workers is necessary and welcomed. We only examined the difference between an on-line fire training program and a control without any training. Therefore, we do not know which mode of delivery is best for fire safety training. We found that approximately 85% of our participants preferred live demonstrations. To this end, studies comparing live demonstrations with on-line video should be carried out in the future. It is likely that on-line videos and live demonstrations have similar effectiveness in delivering knowledge of fire prevention and evacuation, yet on-line video training may still be preferred because it reduces time cost and has better flexibility. The average post-test score was 31.77 out of the total possible score of 45, reflecting considerable room for improvement. It is possible that combining different delivery methods could further improve the level of knowledge of fire prevention and evacuation.

Our study has some limitations and shortcomings. Due to the nature of the study, participants could not be blinded, and they would know their group allocation once they viewed the on-line video. The study was conducted in only five hospitals in China, and our results may not be generalizable to all healthcare workers in China. In particular, all five participating hospitals were provincial- or national-level hospitals located in urbanized cities, and our sample contained relatively few doctors, therapists, and healthcare workers aged 40 or above. It is a common practice to organize fire drills every six months or once per year to reinforce the knowledge of the participants. Our on-line training can provide such a function easily, but its effect should be examined. The long-term effects of on-line knowledge of fire prevention and evacuation training should also be examined. The effectiveness of a training program depends on the study ability of the participants. However, no relevant information about their study ability was collected. Finally, time spent on training reduces the time spent on patient care, which may affect the quality of care. However, we could not assess such an effect as the quality of care was not measured in this study.

Many on-line training programs for healthcare workers had been developed in recent years, and most of them showed promising results with strong effects compared with traditional face-to-face trainings or manual-reading trainings [14, 34, 35]. Of course, on-line training may not always be the best option. In particular, compliance is a major concern for most on-line training programs. The compliance was 100% in the current study as the participants were only required to watch a video of 5 minutes (control) to 15 minutes (intervention) in duration. However, for longer training, it is expected that the compliance will drop drastically. In a series of online workshops (all consisting of a number of modules) offered by the National Center for Suicide Prevention Training in 2002–2004, only 25–45% completed both the pre-test and the post-test [9]. Improving compliance with on-line training should be an important issue in further research. In terms fire training, a virtual environment training that simulated an environment with fire would be more effective in improving participants’ navigation of rescue routes [7] than an on-line training.

To conclude, participation in an on-line fire training program by watching educational video can effectively improve healthcare workers’ knowledge of fire prevention and evacuation. Further research focusing on fire drill and evacuation training in wards with specific needs, for example, obstetrics and gynecology [36, 37] and intensive care units [26], are warranted.

## Supporting information

S1 Fig Screenshots of the on-line training website. (DOCX)

S1 File Study protocol. (DOCX)

S2 File CONSORT 2010 checklist. (DOC)

S3 File Translated questionnaire (in English). (DOCX)

S4 File Minimal anonymized dataset. (SAV)
