# Supplementary material for: The effectiveness of an on-line training program for improving knowledge of fire prevention and evacuation of healthcare workers: A randomized controlled trial
Source: PLoS One. 2018 Jul 5;13(7):e0199747. doi: 10.1371/journal.pone.0199747 (PMC6033414; doi:10.1371/journal.pone.0199747)

Informed consent


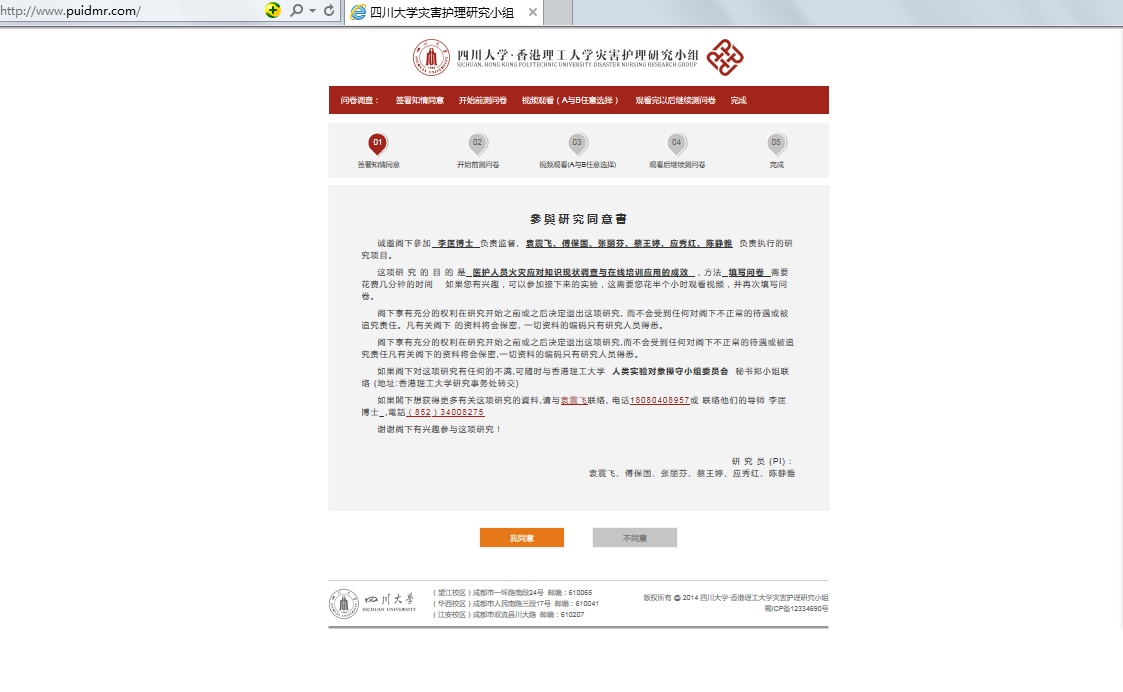


Demographic characteristics


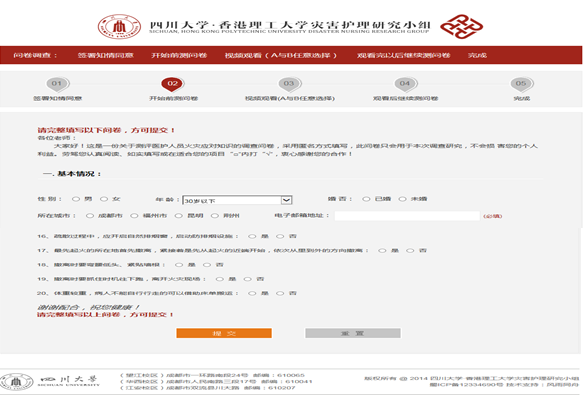


Randomization


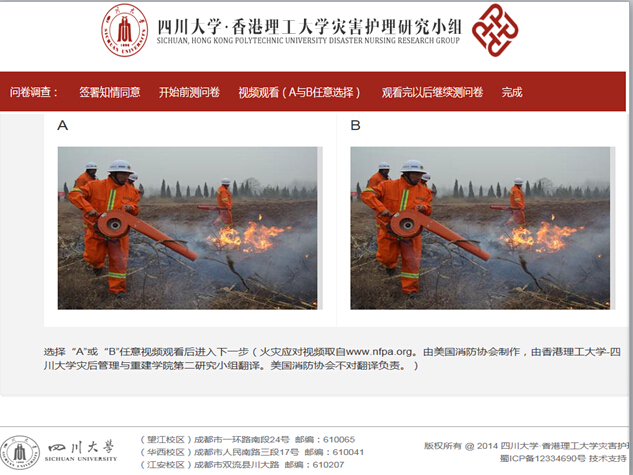


Fire knowledge questionnaire


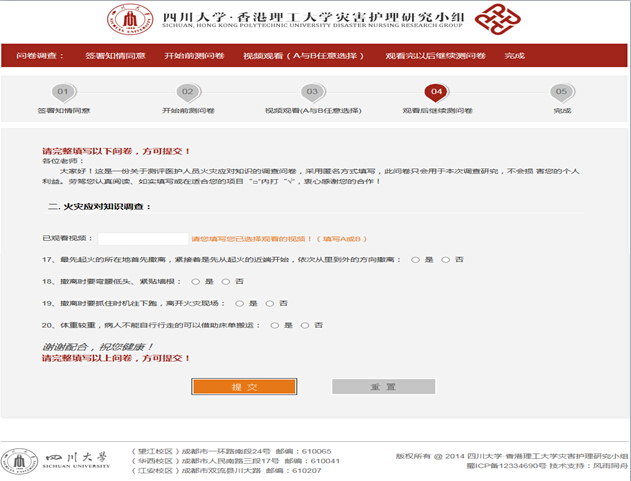

Supplement: S1 Fig — (DOCX) [file pone.0199747.s001.docx]
