# Supplementary material for: The effectiveness of an on-line training program for improving knowledge of fire prevention and evacuation of healthcare workers: A randomized controlled trial
Source: PLoS One. 2018 Jul 5;13(7):e0199747. doi: 10.1371/journal.pone.0199747 (PMC6033414; doi:10.1371/journal.pone.0199747)
Supplement: S1 File — (DOCX) [file pone.0199747.s002.docx]

The effectiveness of an on-line training program for improving fire knowledge of healthcare workers: a randomized controlled trial

**Background:** In recent years, many serious hospital serious fire accident occurred, Murphy and C. Foot (2011) reported that in the UK, about 500 cases of hospital fire occurred every year. The hospital is a special public place, there are "four many" features: many buildings, many people, vulnerable groups - many patients, and many flammable items. The hospital offer 24 hours consultation, many people flow and mobility, and the hospital equipment, numerous, large power consumption, oxygen, ethanol and other flammable and explosive materials, distributed throughout the hospital, in the event of fire, are easy to contribute to the fire, and is bound to cause significant property damage and casualties. Murphy and C. Foot (2011) reported that in the UK, the average annual hospital fire caused about 65 people injured, 1-2 people died. In December 15, 2005, in a fire in Liaoyuan City Central Hospital of Jilin Province, 39 people died, 89 people jumped, led to direct economic losses of about 30 million, this event caused the Health Commission (former Ministry of Health) attaches great importance. Hospital fires may occur at any time, for more reasons, Du Shou-liang (2007) pointed out that due to hospital electrical violations of discipline led to the fire in the Liaoyuan City.

Xu Xia and Zhang Yan (2014) pointed out that although the hospital no smoking signs can be seen everywhere, but cannot prevent the occurrence of smoking; the hospital has large flow of people, the vast majority of them are vulnerable groups, mobility, with evacuation difficulties; medical staff and patients with fire safety awareness is not strong; lack of self-help common sense, in the event of fire, easy to know what to do, more prone to stampede accidents, resulting in casualties, so the hospital fire safety has always been the focus of fire work. The American Fire Protection Association International Exchange Department (2006) an analysis of fire hospital showed that the tragedy of the people to bring the warning is: emergency situations in the fire situation is very much in our country, Wei Planning Commission (2006) stated that "Medical and health institutions disaster prevention and emergency response guidance", in the emergency response to the disaster accident, all levels of medical and health institutions to evacuate, transfer, emergency rescue as a prominent focus. The maximum possible to avoid and reduce casualties. Ministry of Public Security (2010) stated that "project, put forward the social units" to improve the four capabilities "requirements, namely: to check the ability to eliminate fire hazards, the organization to fight the initial fire capacity, the organization evacuation ability, evacuation education and training capacity. Light (2006) compared China and Japan 11 hospitals fire plans and other relevant information, get the following conclusion: hospital fire plans must be detailed, from the staff, materials, exercise training, publicity and education, and other aspects of the development of detailed and careful measures, and must be put into action. The hospital itself has a particular nature, the evacuation of personnel when the fire is very difficult, to some extent, the evacuation itself may cause harm to the patient. Therefore, the hospital must develop practical contingency plans, pre-fire safety measures for patients and repeated training exercises, so that every staff can be proficient in. Only in this way, in order to maximize the protection of patients in the emergency time of life safety. The health care workers are the most direct implementation of the hospital fire plan, but also the process of treatment of patients, the most exposure to the role, so to strengthen the firefighting training of health care workers to improve health awareness of health care workers, is imperative. Dimeff, Koerner and Woodcock (2009) compared the written response manual, the two-day lectures and multimedia interactive online training, the three methods used in the training of clinician dialectical behavioral therapy skills, the results show that, online training effect is significantly better than the traditional training effect. Network training has a strong flexibility, and now with the advent of the Internet age, the network more and more in-depth our lives, the number of applications of the network continues to increase, the network platform training courses are numerous.

**Literature review:** We search the database PubMed, CINAHL, CNKI, Viking database, Wanfang database, search for the key words "fire response knowledge", "health care workers", "online training", "fire response knowledge", "medical personnel", "online training", there are 15 literatures on the hospital 's fire risk. There are five articles on the fire safety of the hospital. The training of the fire in the hospital is based on the training of the hospital. There are 8 articles on the training of medical personnel disaster, 6 articles on online training. After careful reading, we have come to the conclusion that the hospital staff is intensive, the mechanical equipment is large, the load is big , The flammable items, the rapid spread of fire and the hospital's construction and so on constitute the hospital's disaster factors, the hospital more types of disasters, medical personnel disaster training, a single way, training frequency is low, the hospital for fire training, mostly the use of missionary, examinations and the development of plans, resulting in health care workers awareness of fire safety is not strong, lack of self-help and save his knowledge, Online training has a flexible learning time, easy access to learning content, the cost is not high characteristics from the domestic and foreign survey, for the hazards of fire, the world has been a common consensus, and countries have a certain amount of training against fire prevention and response, but the training is mainly for ordinary people's simple fire knowledge lectures, for health care workers to deal with the status quo of fire is unknown, the above, but the way online training almost There is little training on fire knowledge and no clear effect, and there is little research on the use of online training fire knowledge in the world, and there is no basic domestic knowledge.

**Study objective:** To understand the situation and requirements of health care workers' knowledge of fire care and to provide a reference for organizing and conducting fire-related training for special occupational groups such as health care workers, so as to explore how to apply online training to health care workers The necessity and feasibility of the relevant training.

**Objective:** 1) To understand the current situation of fire response knowledge of health care workers in Sichuan, Yunnan, Hubei and Fujian, China; 2) To understand the degree of knowledge of medical staff after handling online training; 3) to test the effect of online training.

**Research question:** 1), the fire training is feasible and effective; 2) online training after the medical staff to deal with the knowledge of the degree of fire has improved; 3) the online training will be applied to the health care workers

**Research significance:** 1) at present, at home and abroad specifically for health care workers hospital fire response knowledge and emergency response capacity of the investigation is very little, less relevant knowledge training, the main investigation of the health care workers on the current situation of fire response knowledge and do online. The results of the training for the results of the hospital for fire prevention and disaster prevention training to provide a reference, so as to ultimately be able to effectively protect the safety of patients. 2) according to the sample of the basic information, such as: title, age, etc., through demographic analysis, Identify the groups of health care workers who need to be trained.

**Methods:** All volunteers signed the consent approved by the Hong Kong Polytechnic University Ethics Committee before the study and the Hong Kong Polytechnic University School of Nursing developed the "Participation in Research Consent". The use of convenient sample survey Sichuan, Yunnan, Hubei, Fujian four provinces three public hospitals and other health care workers on the hospital fire training needs. For the sake of sincerity, the research assistant describes the entire research process in person and invites interested volunteers to participate in the whole process, by e-mail or telephone to keep in touch. Based on our existing guidelines, we have created a questionnaire of about 40 objective multiple choice questions, including the general situation of health care workers and the questionnaire on fire-related knowledge. Including the basic knowledge of fire, emergency response measures, fire escape routes, the use of fire extinguishers and methods of transfer of patients in five areas. The above five related content online training, training, an online lecturer to play with the relevant video, so that volunteers can use a more vivid understanding of the contents of the lecturer.

The data were randomly divided into experimental group and control group. The experimental group was trained on-line training fire knowledge, and the control group was trained on the basis of fire knowledge other than fire knowledge. Before the training, the experimental group and the control group answered the same content Questionnaire. The effectiveness of online training is determined by comparing the vertical and horizontal analysis of the results of the two sets of pre-test and post-test questions.

**Research places:** Sichuan, Yunnan, Hubei, Fujian four provinces five different three comprehensive hospital.

**Subjects:** All subjects were hospitalized frontline clinical medical staff, medical technicians and logistics staff except. (Dimeff, et al., 2009) Similar studies included 25 samples from the experimental and control groups, and the initial sample size was 125.

**Process:** At the baseline assessment, the volunteers voluntarily signed the "participation in the study consent", participated in the study, and informed the final findings. Research materials include network platform, user name and online training password. The evaluation procedure after training depends on the experimental conditions. On the basis of the need, the assistant researcher evaluates the research and encourages the volunteers to complete the training within the prescribed time by telephone, QQ message or e-mail. Follow the principle of confidentiality, to ensure that volunteers will not disclose personal information, respect for volunteers to launch their own rights. When the volunteers completed all the questionnaires, the volunteers in the control group were able to continue the training on the fire. All the volunteers in the experimental group and the control group were able to receive the small number of the individuals we sent within ten days of the completion of the study Gift to show thank you.

**Measurement tool:** Self-developed questionnaires based on existing guidelines.

**Data analysis:** SPSS 19 software.
