# Supplementary material for: The effectiveness of an on-line training program for improving knowledge of fire prevention and evacuation of healthcare workers: A randomized controlled trial
Source: PLoS One. 2018 Jul 5;13(7):e0199747. doi: 10.1371/journal.pone.0199747 (PMC6033414; doi:10.1371/journal.pone.0199747)
Supplement: S3 File — (DOCX) [file pone.0199747.s004.docx]

**Questionnaire**

Hello everyone! This is a questionnaire to assess fire prevention knowledge of healthcare workers. Anonymous forms are used. This questionnaire will only be used in this research and will not harm your personal interests. Please read carefully and fill in the response or ticking "√" in the □ next to your choice. **We sincerely thank you for your cooperation!**

**First part basic situation：**

Gender：□Male □Female Age： years

Marital status：□Married □Single Location / city： 。

Email address： 。

Work experience ：□within 3 years； □≥3 years，≤5 years；□more than 5years，less than or equal to 9 years；□more than 9 years

Title：□Junior □Intermediate □Deputy senior □Senior

Position：

Department：□Internal medicine □Surgery □Obstetrics and Gynecology □Pediatric □Intensive care unit □Operating room □Accident and emergency department □General □Administration □Others： 。

月薪：□1000-3000 □3001-5000 □5001-8000 □8000以上

Religion：□Yes（ ）□No

1. Education level：□Secondary □College □Bachelor □Master □Doctor

2. Had you experienced fire：□Yes □No

3. Has your hospital experienced a fire in the past 3 years：□Yes □No （skip to Question number 5 if you choose "No"）

If yes, the number of occurrences： 。

4. Were there any casualties：□Yes □No

If yes, the number of casualties： 。

5. Have you participated in the fire prevention training organized by your hospital within three months：

□Yes（ times） □No

6. Have you participated in the fire evacuation training organized by your hospital within three months： □Yes □No

7. What is your perceived fire knowledge level：

□Very good □Good □Neutral □Poor □Very poor

8．Do you think fire prevention training is important：

□Very important □Important □Neutral □Unimportant □Very important

9. What is your preferred mode of training：

□Face-to-face □Online □Live demonstration □Manual-reading

Please list out the reason： 。

10. Will you identify the location of the safe passage when entering an unfamiliar place? □Yes □No

11.Have your hospital has fire prevention protocol： □Yes □No

**Second part Generic fire knowledge**

1. When a fire occurs, you should take the nearest elevator to evacuate □Yes ☑ No

2. Cover your nose and mouth with a wet towel when a fire breaks out ☑ Yes □No

3. When a fire breaks out, a large amount of smoke and poisonous gas will be generated ☑ Yes □No

4. If you are at a fire, you can always find the safety exit

☑ Yes □No

5. Do you feel panicked during a fire? □Yes ☑ No

6. Should you rush out a fire? □Yes ☑ No

7. Can you make your own safety rope during a fire to escape? ☑ Yes □No

8. Are you familiar with the sound of fire alarm? ☑ Yes □No

9. Are you familiar with your department's escape route? ☑ Yes □No

10. In an emergency situation and in a high-rise building, will you escape by jumping?

□Yes ☑ No

11. Do you know the fire equipment in your working area? ☑ Yes □No

12. Do you know the function of fire extinguisher? ☑ Yes □No

13. Do you know the procedure of using a fire extinguisher? ☑ Yes □No

14. Do you know the use of emergency lights in a fire emergency? ☑ Yes □No

15. The initial stage of fire is the more unfavorable phase of fire fighting □Yes ☑ No

16. You believe most deaths in a fire belong to being burnt. □Yes ☑ No

17. When using fire extinguishers, they should be targeted at flame position. □Yes ☑ No

18. When there is smoke caused by fire, we should stand upright to escape □Yes ☑ No

19. The key to escape path should be transferred to the next shift and put in a fixed place □Yes ☑ No

20. Do you know the role and location of smoke detectors? ☑Yes □No

21. Did you know the function and location of the sprinkler system? ☑ Yes □No

22. Do you know the fire door is closed? ☑ Yes □No

23. Do you know the escape floor is at the top floor? □Yes ☑ No

24. What do you think is the most important thing in a fire?

☑A．Dial 119 B. Remain calm and confident

C．Follow other people D. Find places to escape

25. If you are trapped in the fire, which of the following should you do?

①wrapped yourself with wet clothes ②cry our loud for others to locate yourself；

③lower your body, cover your mouth and nose； ④rush out of smoke；

A. ①②③④ ☑B. ①②③ C. ①③④ D. ①②④

**Third part Hospital-specific fire knowledge**

1. Do you know how to help patients who could not walk to leave the scene of the fire? ☑ Yes □No

2. Do you know how to help patients having intravenous infusion to escape? ☑ Yes □No

3. Do you know how to move patients who cannot move independently alone? ☑ Yes □No

4. Do you know how to evacuate patients who need continuous oxygen support? ☑ Yes □No

5. Dry powder fire extinguishers should be used to put off fire on electronic equipment □Yes ☑ No

6. Doors and windows should be kept open and ventilated when caught in a relatively closed ward □Yes ☑ No

7. Patients’ property should be picked up during fire □Yes ☑ No

8. Foam fire extinguisher should be used for alcohol-burned fires。 ☑ Yes □No

9. If a fire is found, all personnel should be immediately evacuated □Yes ☑ No

10. If a fire is found, the security should be notified, fire hydrants should not be used without permission □Yes ☑ No

11. When the fire is serious, keep the window ventilated to avoid dust poisoning □Yes ☑ No

12. When the fire is serious, the corridor lights should be switched on to ensure the smooth evacuation process ☑ Yes □No

13. When using fire extinguishers, we should stand in the upwind position and keep a safe distance with the fire ☑ Yes □No

14. When using a dry powder fire extinguisher, we should wear gloves to prevent frostbite □Yes ☑ No

15. During fire evacuation, wet towels should be used to cover your nose and mouth to prevent inhalation of toxic gases ☑ Yes □No

16. During fire evacuation, natural smoke windows should be opened to extract the smoke □Yes ☑ No

17. The position where the first fire started should be evacuated first, followed by the proximal position and subsequently from inside out ☑ Yes □No

18. During fire evacuation, we should bow down and keep close to the wall ☑ Yes □No

19. During fire evacuation, we should seize the opportunity to run down and leave the fire □Yes ☑ No

20. For overweight patients and bed-bound patients should be evacuated by bed linen ☑ Yes □No
